# Fever Unveiling a Hidden Cardiac Condition: A Case of Pediatric Hypertrophic Obstructive Cardiomyopathy

**DOI:** 10.7759/cureus.55823

**Published:** 2024-03-08

**Authors:** Sneha Reddy, Ashish Varma, Amar Taksande

**Affiliations:** 1 Pediatrics, Jawaharlal Nehru Medical College, Datta Meghe Institute of Higher Education and Research, Wardha, IND

**Keywords:** multidisciplinary management, beta-blocker therapy, echocardiography, fever, hypertrophic obstructive cardiomyopathy (hocm), pediatric

## Abstract

This case report presents the clinical management of a 18-month-old female child who presented with fever, cough, and cold symptoms for eight days. Despite initial treatment with antipyretic syrup, the persistence of symptoms prompted further evaluation, revealing signs of hypertrophic obstructive cardiomyopathy (HOCM) on echocardiography. The patient was subsequently initiated on beta-blocker therapy and supportive care, leading to clinical improvement and eventual discharge. This case underscores the importance of considering cardiac etiologies in pediatric patients presenting with nonspecific symptoms. It highlights the role of timely diagnosis and multidisciplinary management in optimizing outcomes for affected individuals. Further research and awareness efforts are warranted to enhance diagnostic capabilities and refine treatment strategies for pediatric cardiac conditions like HOCM.

## Introduction

Hypertrophic obstructive cardiomyopathy (HOCM) is a primary cardiac disorder characterized by asymmetric hypertrophy of the myocardium, particularly involving the interventricular septum, leading to dynamic obstruction of the left ventricular outflow tract (LVOT) [1]. This condition is rare in pediatric populations but can present with diverse clinical manifestations, including fever and cough [2]. The diagnosis of HOCM often relies on echocardiography, which reveals signs of asymmetrical septal hypertrophy and LVOT obstruction, as well as associated valvular abnormalities such as mitral regurgitation (MR) and pulmonary stenosis (PS) [3]. Management strategies for pediatric patients with HOCM typically involve medical therapy aimed at alleviating symptoms and reducing obstruction. Beta-blockers, such as Propranolol, are commonly prescribed to mitigate LVOT obstruction and improve clinical outcomes [4].

In cases where medical therapy is inadequate, surgical interventions such as septal myectomy or alcohol septal ablation may be considered to alleviate LVOT obstruction and improve cardiac function [5]. Prompt recognition and management of HOCM are crucial to prevent complications such as heart failure, arrhythmias, and sudden cardiac death in affected pediatric patients [6]. This case report highlights the importance of considering cardiac etiologies, including HOCM, in the differential diagnosis of pediatric patients presenting with nonspecific symptoms such as fever and cough. Timely diagnosis and appropriate management are essential to optimize outcomes and prevent complications in affected individuals.

## Case presentation

**Figure 1:**
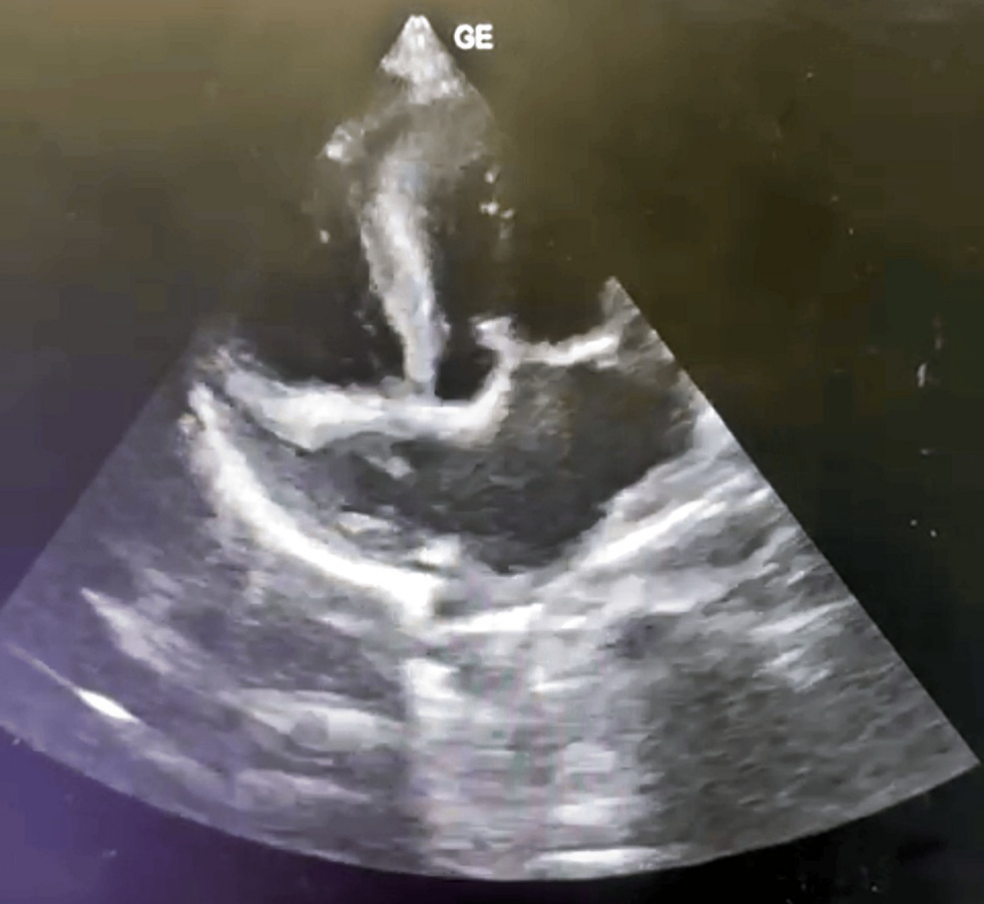
Shows indications of asymmetrical septal hypertrophy and a peak gradient in the left ventricular outflow tract (LVOT) measuring 96 mmHg, suggestive of hypertrophic obstructive cardiomyopathy (HOCM)

An 18-month-old female child was brought by her parents to the pediatric outpatient department with complaints of fever, cold, and cough persisting for the past eight days. Upon obtaining the medical history, it was disclosed that the child had previously sought treatment at a local private hospital, where she was managed with antipyretic syrup containing Paracetamol. Following a suggestion, a 2D echocardiogram was performed, revealing indications of asymmetrical septal hypertrophy and a peak gradient in the LVOT measuring 96 mmHg, suggestive of HOCM (Figure 1). According to the mother's account, the child was asymptomatic eight days prior before developing a fever of insidious onset that persisted despite medication. The fever was concomitant with cough and cold symptoms, occasionally leading to non-projectile post-tussive vomiting (2-3 episodes within the past eight days). No complaints of loose stools or distress were reported.

Upon physical examination, vital signs indicated an elevated body temperature of 100.70 Fahrenheit, a pulse rate of 128 beats per minute, a respiratory rate of 28 breaths per minute, and an oxygen saturation level of 97%. The child exhibited warmth upon touch, prompting admission to the tertiary care hospital in central India. Upon admission, the child was initiated on intravenous administration of cefotaxime, amikacin, pantoprazole, vitamin D3 drops, and syrup Paracetamol.

Blood samples were obtained and sent for laboratory tests, including a complete blood count, which revealed a hemoglobin level of 7.7, a total leukocyte count of 13180, and a platelet count of 4.41. Kidney function tests and liver function tests were also conducted and found to be within normal limits. A chest X-ray demonstrated evidence of cardiomegaly. A thyroid profile was also conducted due to coarse facial features, yielding normal results.

**Figure 2:**
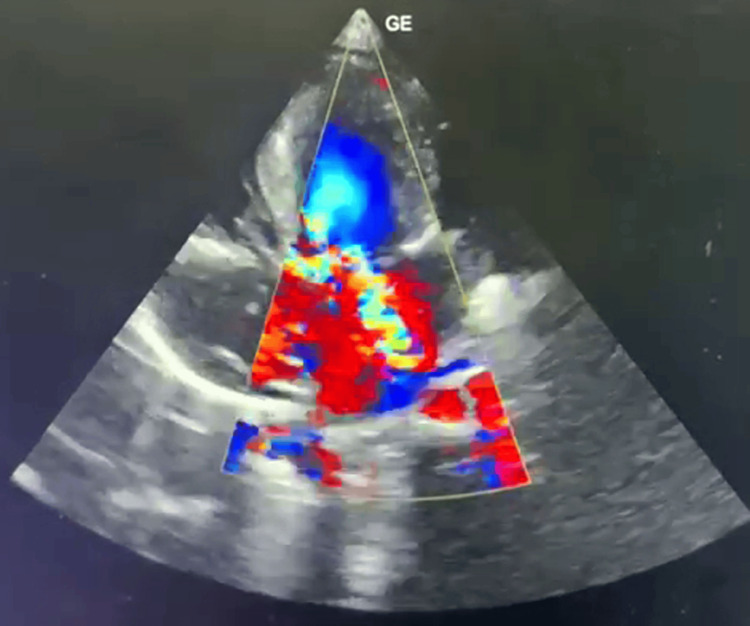
Confirmed asymmetrical septal hypertrophy, a peak gradient in the LVOT of 96 mmHg, indicative of HOCM, along with moderate to severe mitral regurgitation (MR), moderate valvular and supravalvular pulmonary stenosis (PS), and a prominent eustachian valve

A cardiology consultation was sought, and the 2D echocardiographic findings confirmed asymmetrical septal hypertrophy, a peak gradient in the LVOT of 96 mmHg, indicative of HOCM, along with moderate to severe MR, moderate valvular and supravalvular PS, and a prominent eustachian valve (Figure 2). The attending physician prescribed the tablet Propranolol at 1 mg/kg once daily. The child exhibited bilateral conducted sounds and cough, prompting initiation of nebulization with 3% NaCl and Kufril LS drops.

After three days, intravenous antibiotics were discontinued, and the child was transitioned to syrup Codon. With stable vital signs and hemodynamics, the child was discharged with instructions for follow-up care.

## Discussion

The presented case underscores the importance of recognizing cardiac etiologies, particularly HOCM, in pediatric patients presenting with nonspecific symptoms such as fever and cough. While HOCM predominantly affects adults, its occurrence in pediatric populations is rare yet significant [6]. This case highlights the need for a comprehensive diagnostic approach to elucidate the underlying cause of symptoms in pediatric patients, especially when initial presentations are atypical.

The diagnosis of HOCM in this case was facilitated by echocardiographic findings demonstrating asymmetrical septal hypertrophy and a significant peak gradient in the LVOT, consistent with previous literature [7]. Additionally, associated abnormalities such as MR, valvular and supravalvular PS, and a prominent eustachian valve were observed, further supporting the diagnosis [8]. Using echocardiography as a diagnostic modality aligns with current guidelines and recommendations for evaluating pediatric patients with suspected cardiac abnormalities [9].

Medical management of HOCM in this case consisted of initiating beta-blocker therapy with Propranolol, consistent with established treatment strategies aimed at reducing LVOT obstruction and alleviating symptoms [10]. Furthermore, supportive measures, including nebulization and symptomatic relief with analgesics, were employed to address concurrent symptoms such as cough and fever. Deciding to discontinue intravenous antibiotic therapy after three days and transition to oral medication reflects a prudent approach to antimicrobial stewardship, minimizing the risk of adverse effects associated with prolonged intravenous therapy without compromising clinical outcomes [11].

Although surgical interventions such as septal myectomy or alcohol septal ablation were not pursued in this case, they remain viable options for patients with refractory symptoms or severe LVOT obstruction unresponsive to medical therapy [12]. Surgical interventions may offer long-term benefits in select pediatric patients with HOCM, particularly those with significant functional impairment or high-risk features [13].

## Conclusions

In conclusion, the presented case underscores the importance of considering HOCM as a potential cause in pediatric patients exhibiting nonspecific symptoms such as fever and cough. The underlying cardiac abnormality was promptly identified through diligent diagnostic evaluation, including echocardiography, facilitating timely medical intervention with beta-blockers and adjunctive supportive care. This case highlights the necessity for prompt recognition and management of cardiac conditions in pediatric populations, as delayed diagnosis and treatment may lead to significant complications. Furthermore, it emphasizes the essential role of interdisciplinary collaboration between pediatricians and cardiologists to ensure comprehensive care and optimized outcomes for affected individuals. Continued research and heightened awareness regarding HOCM in pediatric patients are crucial for refining diagnostic strategies and therapeutic approaches, ultimately enhancing patient prognosis and quality of life. By sharing and discussing cases like this, healthcare professionals can contribute to advancing the understanding and management of cardiac disorders in pediatric populations, ultimately improving patient outcomes and long-term health.
